# On the Relation of the Nervous System to Disease and Disorder in the Viscera

**Published:** 1900-03

**Authors:** 


**On the Relation of the Nervous System to Disease and Disorder in the Viscera.** By Alexander Morison, M.D. Pp. xiv., 132. Edinburgh: Young J. Pentland. 1899.

The Morison lectures delivered before the Royal College of Physicians in Edinburgh in 1897 and 1898 form the groundwork of this volume, which deals with the histology of the visceral nervous system, more especially as revealed by modern investigations by Golgi's process of staining, and afterwards with the pathology of disorders of visceral motion and sensibility.

There is but little evidence of original work in the book, and the discussion of the various problems is not very illuminative or suggestive. It must be confessed that one does not rise from reading the volume with much clearer ideas on the subject.

This is perhaps inevitable; for as the histology and physiology of the visceral nervous system are hardly known, the pathology scarcely admits of generalisations.

